# An Operational Framework for Insecticide Resistance Management Planning

**DOI:** 10.3201/eid2205.150984

**Published:** 2016-05

**Authors:** Emmanuel Chanda, Edward K. Thomsen, Mulenga Musapa, Mulakwa Kamuliwo, William G. Brogdon, Douglas E. Norris, Freddie Masaninga, Robert Wirtz, Chadwick H. Sikaala, Mbanga Muleba, Allen Craig, John M. Govere, Hilary Ranson, Janet Hemingway, Aklilu Seyoum, Michael B. Macdonald, Michael Coleman

**Affiliations:** Ministry of Health, Lusaka, Zambia (E. Chanda, M. Kamuliwo, C.H. Sikaala);; Liverpool School of Tropical Medicine, Liverpool, UK (E.K. Thomsen, H. Ranson, J. Hemingway, M. Coleman);; Abt Associates, Lusaka (M. Musapa);; Centers for Disease Control and Prevention, Atlanta, Georgia, USA (W.G. Brogdon, R. Wirtz, A. Craig);; Johns Hopkins University Bloomberg School of Public Health, Baltimore, Maryland, USA (D.E. Norris);; World Health Organization, Lusaka (F. Masaninga);; Tropical Disease Research Centre, Ndola, Zambia (M. Muleba);; University of the Witwatersrand, Johannesburg, South Africa (J.M. Govere);; Africa Indoor Residual Spraying Project, Accra, Ghana (A. Seyoum);; Consultant, Baltimore (M.B. Macdonald)

**Keywords:** arthropod vectors, insect vectors, insecticide resistance, insecticides, malaria, lymphatic filariasis, pest control, policy making, parasites, mosquitoes, vector-borne infections, Zambia

## Abstract

Arthropod vectors transmit organisms that cause many emerging and reemerging diseases, and their control is reliant mainly on the use of chemical insecticides. Only a few classes of insecticides are available for public health use, and the increased spread of insecticide resistance is a major threat to sustainable disease control. The primary strategy for mitigating the detrimental effects of insecticide resistance is the development of an insecticide resistance management plan. However, few examples exist to show how to implement such plans programmatically. We describe the formulation and implementation of a resistance management plan for mosquito vectors of human disease in Zambia. We also discuss challenges, steps taken to address the challenges, and directions for the future.

## Abstract 2

Country-wide planning and coordination can improve sustainability of vectorborne disease control.

Emerging and reemerging infectious diseases are often transmitted by arthropod vectors (*1**,**2*). A primary strategy to reduce vectorborne disease transmission is the use of insecticides for public health. However, resistance to insecticides has appeared in all major insect vectors of human disease (*3*) and has rapidly increased in prevalence and intensity over the past decade (*4*). Insecticide resistance is worrisome, in part because it has repeatedly been implicated as a cause of disease resurgence, particularly for malaria (*5**–**8*).

Although the spread of resistance in a population of organisms challenged by a drug or insecticide is inevitable, the public health community has not yet taken the steps necessary to safeguard the limited number of insecticides available. Consequently, their continued efficacy is at risk (*9*), setting the stage for reemergence of vectorborne diseases in locations where insecticide-based control measures are implemented. In response to this concern, the World Health Organization published the Global Plan for Insecticide Resistance Management (GPIRM) in 2012 (*10*). This document provides vectorborne disease control programs and other stakeholders with a strategic direction for approaching the resistance crisis.

The GPIRM outlines the need for increased resistance monitoring, data management capacity, and implementation of strategies to manage insecticide resistance. The responsibility for implementing this management plan lies with disease-endemic countries, with support from their global partners. Among the challenges in carrying out these recommendations are lack of options for insecticide rotations (the primary strategy for curbing spread of resistance), inconsistent resistance monitoring procedures, reluctance to share resistance data, and lack of data management capacity in disease-endemic countries (*11*). In addition, a reliance on donor funding, which can be unpredictable, threatens the sustainability of enacted plans.

By 2014, only a few countries had established insecticide resistance management plans (IRMPs) and incorporated them into operational malaria vector control programs (*11*). However, no country has documented how it formulated or executed these policies and addressed challenges. To realize fully the vision of the GPIRM, national malaria control programs must share experiences regarding policy-making processes. We report on the formulation and implementation of a new insecticide resistance management plan in Zambia during 2009–2014.

## Local Setting

Because of the increased political commitment since the Abuja Declaration in 2001, funding for malaria control has increased (*12*). The widespread use of insecticide-based vector control subsequently reemerged in many malaria-endemic countries, including Zambia. In 2000, the private sector reintroduced indoor residual spraying (IRS) with 2 classes of insecticides, DDT and pyrethroids, in 2 districts in Zambia’s Copperbelt province (*13*). The success of these IRS programs led Zambia’s National Malaria Control Centre (NMCC) to implement IRS and distribution of long-lasting insecticidal nets (LLINs) (*14*). IRS use was scaled up from 5 districts in 2003 to all 72 districts by 2011 (*15*) (the total number of Zambia districts increased to 105 in 2013). Countrywide mass distribution of LLINs has occurred since 2005; currently, 72% of households own >1 LLIN (*16*). For 9 years after IRS was introduced in 2000, vector control relied exclusively on 2 insecticide classes with 1 mode of action (i.e., both DDT and pyrethroids target the voltage-gated sodium channel of nerve cells to cause death of the insect). However, in 2009, resistance to both insecticide classes was detected in the major malaria and lymphatic filariasis vectors (*17*).

**Figure 1:**
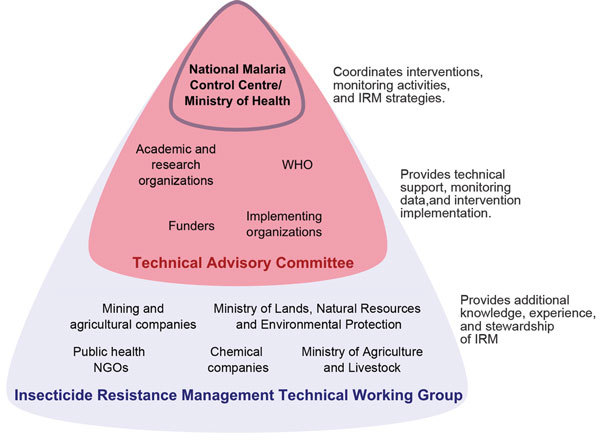
Composition of the Insecticide Resistance Management (IRM) Technical Working Group and the Technical Advisory Committee in Zambia and roles of member organizations. NGOs, nongovernment organizations; WHO, World Health Organization.

After this resistance was detected, the NMCC formed the Insecticide Resistance Management Technical Working Group (IRMTWG) in 2010. This group provides a foundation for the policy development process by coordinating policy formulation, implementation, and evaluation. The IRMTWG is multisectoral and comprises members of government, nongovernment, and private organizations having a vested interest in vector control (Figure 1). A subset of IRMTWG members forms the Technical Advisory Committee (TAC), which aids the NMCC in interpreting the results of the implementation process and provides recommendations about the most appropriate actions. The NMCC serves as the secretariat of the IRMTWG and coordinates the implementation of entomologic surveillance and resistance monitoring in the country.

In April 2011, through a series of conference calls, the IRMTWG defined the following policy objectives: 1) gather more phenotypic resistance data through partners serving as channels of information for different parts of the country, and establish a sustainable monitoring protocol; 2) determine the underlying mechanisms involved in resistance of all the major vectors in Zambia; 3) establish and maintain a database for all insecticide resistance data; and 4) develop an IRMP informed by data and agreed on by all stakeholders. The group recognized that to achieve the last objective, objectives 1–3 must be first realized. Therefore, implementation of the policy was envisioned as a multiphased process.

## Objective 1: Expand Resistance Data

Although initial data presented to the incipient IRMTWG were limited, they highlighted insecticide resistance in Zambia, in particular, DDT resistance in areas where this insecticide was used (*17*). In addition, 2 obstacles became apparent. First, the available data revealed only a small portion of the insecticide resistance in the country, but the data could be expanded through the collaboration of other stakeholders, including research (local and international), private (mining and agricultural), nongovernment organizations, and insecticide companies. Second, even if all partners agreed to share data, a concerted and organized effort to collect more data was necessary.

The formation of the IRMTWG and its inclusion of all stakeholders assuaged the doubts of many of its participants about sharing data. Discussions about data ownership and use continue to the present day, and all participants agree that data-sharing does not prevent publication. In addition, partners are willing to share data because of the NMCC’s role in leading the process. Their willingness to share data might not have occurred if the process were led by an outside organization. With data from the entire country in hand, the IRMTWG and TAC could make informed recommendations about which locations needed additional data and which organization was in the best position to collect that data.

**Figure 2:**
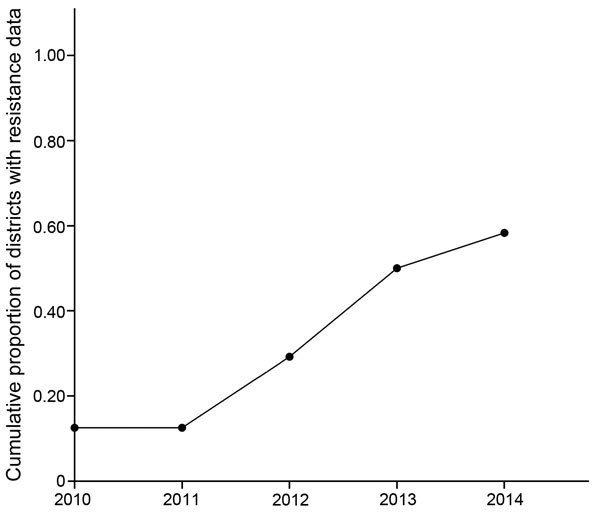
Increase in the number of geographic locations producing data on insecticide resistance in malaria vectors, Zambia, 2010–2014.

As a result of data-sharing and increased monitoring efforts, the cumulative number of geographic foci with resistance data increased at a staggering pace (Figure 2), largely because of efficient interactions between the IRMTWG and policy implementers (i.e., the NMCC and the Zambia Integrated Systems Strengthening Program, supported by the US government’s President’s Malaria Initiative [PMI]). During the annual meeting, the IRMTWG collates and interprets the available resistance data and provides guidance about locations for focusing monitoring efforts for the following year. This work has resulted in a better understanding of the resistance profiles of the major malaria vectors throughout the country.

### Sustained Monitoring and Evaluation

The insecticide resistance profile for many areas of the country was determined, each at a specific time, during 2011–2014. However, 9 sites in districts where IRS was being implemented were selected for longitudinal monitoring of mosquito populations. In these sites, light traps (developed by the US Centers for Disease Control and Prevention [CDC]) and pyrethrum spray catches were used to monitor mosquito density and behavior; insecticide bioassays (*18*) were performed every 6 months to monitor prevalence of resistance. In 2014, the IRMTWG decided that the NMCC should increase the number of sites being monitored from 9 to 24 sentinel sites spread across the country. When resistance is detected by using a diagnostic dose, quantifying the strength of that resistance and determining how the resistance may impact vector control are needed. In Burkina Faso, the strength of resistance increased significantly over just 3 years (*19*), leading to decreased effectiveness of bed nets. Consequently, CDC bottle bioassays (*20*) will be used to measure intensity of resistance in the original 9 sentinel sites.

The primary challenge in implementing this monitoring scheme is the limited financial and human resources that any single organization has to devote to such a large task. This difficulty was alleviated when the NMCC took the lead in the insecticide resistance management process. Although funding comes from different sources (e.g., government of Zambia, World Health Organization, PMI, UK Department for International Development, and the Global Fund), having the NMCC at the helm helps divide monitoring procedures among partners in an organized fashion and facilitates the standardization of monitoring protocols across sites.

## Objective 2: Determine Mechanisms of Resistance

With development of molecular techniques for resistance detection, programs can now detect the presence of resistance genes in vectors and monitor how gene frequencies change over time. Both Equatorial Guinea (*21*) and Zambia (*22*) have shown the value of using rapid molecular assessment of local vectors to inform operational decision-making.

Details of insecticide resistance in Zambia and the means of data collection have been documented (*17**,**22*). Briefly, *Anopheles gambiae* mosquitoes are resistant to DDT and pyrethroids because of the target site mutation *kdr* (knockdown resistance) L1014F and elevated P450 and **glutathione S-transferase** detoxification enzymes; *An. funestus* mosquitoes are resistant to carbamates and pyrethroids because of elevated P450. In *An. funestus* mosquitoes, this resistance mechanism is the same one that led to the failure of the pyrethroid-based control program in South Africa during the 1990s (*23*) and that has been detected in Malawi (*24*) and Mozambique (*25*). These findings greatly influenced the decision-making process surrounding the IRM strategy in Zambia; they confirmed that pyrethroids should no longer be included in insecticide rotations because of the metabolic mechanism of resistance (*10*).

## Objective 3: Establish a Central Data Repository

Reliable and available resistance-monitoring data are essential for evidence-based decision-making. The Disease Data Management System (DDMS) entomology module (*26*) is used for collating the data, and evaluation of the DDMS in Zambia and other countries has been reported (*27*). Historical data since 2011 were imported into the DDMS from legacy systems (typically Excel or Access [Microsoft, Redmond, WA, USA]), and data from ongoing collections have been entered into the system directly. The DDMS accepts all data used by vectorborne disease control programs and generates reports in many formats. When evaluating resistance data, the DDMS has become a valuable tool for the IRMTWG and TAC. Information is more easily collated and mapped than previously and enables more informed decision-making and close monitoring of the IRM policy development process.

The primary challenge in adopting a system like the DDMS has been a lack of data management capacity within the NMCC. Components of entomologic monitoring and evaluation, such as data management and decision support systems like the DDMS, must have allocations of sufficient financial and human resources if they are to realize their full potential in guiding disease control policy. The NMCC and the Zambia Integrated Systems Strengthening Program sought technical assistance for training end users, from data entry to management levels, so that all parties were competent in using the system. The annual IRMTWG meetings that require access to the data and reports generated from the DDMS provide pressure to use the system (i.e., enter and check the data) throughout the year.

## Objective 4: Policy Change and the IRMP

The primary goal of the IRMTWG annual meetings is to interpret resistance data to make informed decisions about which insecticides to deploy for IRS during the next spray season. During 2011–2013, when implementing partners were strategically collecting resistance data to better inform the IRM strategy, the TAC had to make decisions about IRS in Zambia, although some parts of the country were not comprehensively supported by data. In 2011, the first major change to the IRS strategy was implemented: withdrawing DDT and introducing carbamates and organophosphates. By using the resistance data available at the time, TAC decided that carbamates should be deployed in Northern, Muchinga, Luapula, and Copperbelt provinces, whereas organophosphates would be used in Eastern province and pyrethroids in the rest of the country. After the necessary legislative and regulatory approvals were obtained from the Zambia Environmental Management Agency, procedures for procuring these insecticides immediately commenced. Both the PMI and the government of Zambia, which supported the IRS operations, issued bids for contracts, and contending bidders registered with the Zambia Environmental Management Agency as a regulatory compliance procedure for supplying carbamates and organophosphates. To operationalize the new policy, personnel in public and private sectors conducted train-the-trainer workshops and cascade trainings that emphasized safe use, storage, and disposal of carbamates and organophosphates (*28*).

In 2012, the TAC reviewed the latest data from all IRMTWG member organizations and advised the NMCC to rotate organophosphates with carbamates in Northern, Muchinga, and Copperbelt provinces for the 2012 IRS campaign. The same insecticides used in 2011 were recommended in the other provinces because of lack of available options or lack of data to support decisions. Unfortunately, this strategy was not executed, and Northern, Muchinga, and Copperbelt provinces were sprayed with carbamates again in 2012 because of an insufficient amount of the new formulation of the organophosphate pirimiphos-methyl, Actellic 300CS (Syngenta, Basil, Switzerland), being produced by the supplier. In addition, because of the paucity of data in the western half of the country, the TAC advised that efforts should be made to collect resistance data in these areas.

In 2013, efforts to collect resistance data in North-Western and Western provinces were increased to better inform decision-making. The available data indicated that resistance to all classes of insecticides except the organophosphates was present in all areas of Zambia. Therefore, the TAC recommended that the NMCC spray the entire country with the organophosphate pirimiphos-methyl in 2013. However, only Luapula, Northern, Muchinga, and Eastern provinces were sprayed in 2013 because of increased cost. The TAC recommended continued monitoring of resistance across the country, with additional efforts in Luapula province, where resistance patterns to pyrethroids appeared to be inconsistent.

With the resistance profile in all areas of the country established, the insecticide resistance database in use, and regular IRMTWG and TAC meetings being held, the culmination of several years of hard work was evident in 2014. In preparation for the annual IRMTWG and TAC meeting in May, the first official IRMP was drafted and distributed to members in March 2014. Over the course of the meetings, the IRMP was revised and modified, resulting in a living document that will guide the control program about IRM strategies, monitoring and evaluation, and operational research priorities (Zambia Ministry of Health, unpub. data). The plan highlights the necessity of avoiding pyrethroids for IRS because of widespread, metabolically mediated resistance. In addition, it recommends that organophosphates be used in rotation with DDT (where *An. funestus* or *An. arabiensis* mosquitoes are the primary vectors) to control vector populations effectively while simultaneously reducing selection pressure of any specific active ingredient. The plan highlights several knowledge and resource gaps, including the limited number of insecticides available for IRS, the limited human resource and institutional capacity to deliver monitoring and evaluation, and limited data available to better target IRS interventions.

The primary challenge in developing the IRMP has been a lack of options for insecticide rotation. Ideally, insecticides that have different modes of action should be alternated or used in a mosaic to reduce selection pressure. However, only 4 classes of insecticides with 2 modes of action are currently recommended for IRS. The primary vectors in Zambia have different cross-resistance patterns, and their ranges overlap throughout much of the country, making the choice for control limited in an insecticide-based program. Industry must continue to develop new classes of insecticides that can be used in a public health context. The Innovative Vector Control Consortium (*11**,**29*) is a public-private partnership that has made great strides in this endeavor. Another strategy that may lessen selection pressure on a single insecticide is the use of larvicides, for which more options are currently available than for adulticides. If larvicides become an important part of the vector control program in the future, resulting data will need to be incorporated into the IRMP.

## Discussion

The impact of the policy changes (i.e., to alternate insecticides and cease pyrethroid use) on the reversal of resistance, mosquito abundance, and malaria incidence is currently being monitored, and preliminary results are promising. In some areas of Eastern province, before the switch to Actellic 300CS, *An. funestus* sensu lato mosquitoes showed a very high intensity of resistance to deltamethrin (up to 40% survival at 10 times the diagnostic dose by using CDC intensity assays). Currently, after 2 years of organophosphate spraying, preliminary results indicate a substantial reduction in the intensity of resistance to deltamethrin. Because resistance mechanisms are generally considered biologically costly (*30*), deltamethrin resistance may be decreasing in the absence of any strong pyrethroid selection pressure. However, monitoring is needed to confirm the reversal of resistance and to establish the causative mechanism. The effects of the policy on entomologic and epidemiologic parameters are still being monitored; recently increased efforts include quantifying entomologic indicators of transmission throughout sprayed areas and conducting biannual malaria indicator surveys to track disease trends over time.

A key to the success of Zambia’s insecticide resistance management policy development has been the establishment of a multidisciplinary IRMTWG and the expertise of the TAC. These 2 bodies serve as the fulcrum of the entire policy development process by defining objectives, reviewing progress, and actively responding to feedback from policy implementers. This oversight has resulted in effective collaborations among stakeholders and has facilitated accumulation of entomologic data, greater understanding of resistance mechanisms, and establishment of a shared database for insecticide resistance data. However, the success of this policy change relies on continued investment in monitoring and evaluation, industry’s development of insecticides with new modes of action, and the building of capacity and infrastructure so that reliance on donor funding and resources can be lessened in the future. Although sentinel sites for entomologic monitoring increased over time on a national scale, adoption of more cost-effective monitoring schemes, such as community-based surveillance (*31*), is needed to ensure sustainability.

Insecticide resistance will contribute to disease reemergence if not managed appropriately (*5*). Although the malaria control community is currently at the forefront of this issue, other vectorborne infections for which control measures rely heavily on the use of insecticides should be proactively mitigating the effects of insecticide resistance on transmission. Other vectorborne infections that are endemic in Zambia (e.g., lymphatic filariasis and dengue) do not have control programs that implement vector control. However, dengue control in other areas relies extensively on the use of insecticides to control immature stages of insects, and resistance to organophosphates and pyrethroids is widespread in the primary vector *Aedes aegypti* mosquitoes (*32*). The control of triatomine bugs that transmit Chagas disease is primarily accomplished through the spraying of residual insecticides in houses (*33*). However, pyrethroid-resistant vector populations are now widespread (*34*). Visceral leishmaniasis, a disease transmitted by phlebotomine sandflies, is also largely controlled by IRS, but various populations have been found to be resistant to the insecticides used in these applications (*35**,**36*). Resistance monitoring and management must be integrated into all vectorborne disease control programs so that available insecticides can be used judiciously and the efficacy of chemical-based control can be sustained for the long term.
